# The Process of Wrapping Virus Revealed by a Force Tracing Technique and Simulations

**DOI:** 10.1002/advs.201600489

**Published:** 2017-04-26

**Authors:** Yangang Pan, Fuxian Zhang, Liuyang Zhang, Shuheng Liu, Mingjun Cai, Yuping Shan, Xianqiao Wang, Hanzhong Wang, Hongda Wang

**Affiliations:** ^1^ State Key Laboratory of Electroanalytical Chemistry Changchun Institute of Applied Chemistry Chinese Academy of Sciences Changchun Jilin 130022 P. R. China; ^2^ Key Laboratory of Special Pathogens and Biosafety Center for Emerging Infectious Diseases Wuhan Institute of Virology Chinese Academy of Sciences Wuhan 430071 China; ^3^ College of Engineering University of Georgia Athens GA 30602 USA; ^4^ School of Chemistry and Life Science Advanced Institute of Materials Science Changchun University of Technology Changchun 130012 China; ^5^ State Key Laboratory of Electroanalytical Chemistry University of Chinese Academy of Sciences Beijing 100049 P. R. China

**Keywords:** force tracing, HEV71, internalization, single particle simulation, wrapped

## Abstract

Viral entry into the host cell is the first step of virus infection; however, its dynamic process via endocytosis remains largely elusive. Here, the force tracing technique and single particle simulation are combined to investigate the invagination of single human enterovirus 71 (HEV71, a positive single‐stranded RNA virus that is associated with hand, foot, and mouth disease) via cell membranes during its host cell entry. The experimental results reveal that the HEV71 invaginates in membrane vesicles at a force of 58 ± 16 pN, a duration time of 278 ± 68 ms. The simulation further shows that the virus can reach a partially wrapped state very fast, then the upper surface of the virus is covered by the membrane traveling over a long period of time. Combining the experiment with the simulation, the mechanism of membrane wrapping of virus is uncovered, which provides new insights into how the cell is operated to initiate the endocytosis of virus.

## 1 Introduction

Human enterovirus 71 (HEV71), a positive single‐stranded RNA virus belonging to the family of Picornaviridae, is well known as the dominant causative pathogen of hand, foot, and mouth disease (HFMD), as well as severe neurological disorders in some patients, especially infants and children.^1^, ^2^ HEV71 has become a serious public health threat across the Asia‐Pacific region, and joint attention is needed for effective antiviral therapy and vaccine. The pathogenesis of HFMD has been widely reported to be associated with viral infection‐induced cell death.^3^, ^4^ Experimental results verified that HEV71 enters permissive cells via a receptor‐mediated endocytosis. Two receptors for HEV71 have been identified: human P‐selectin glycoprotein ligand‐1 (PSGL‐1) and human scavenger receptor class B member 2 (SCARB2).^3^, ^5^ During this process, the plasma membrane invaginates to form vesicle, which is pivotal for wrapping the virus. Filament growth and membrane deformation generate forces that promote invagination of the virus.^6^ Many simulation studies have focused on this process of endocytosis, and provided useful information for understanding this process.^7^ Cell membranes are considered as the first barrier to defend cells from external harms.^8^ To complete the replicative cycle, virus must penetrate cell membranes. However, the real dynamic parameters for this step, such as forces that generated in the process of endocytosis and the time of wrapping virus by cell membranes, of single‐virus invagination are still unclear. The lack of dynamic parameters of single HEV71 invagination severely hinders the research on HFMD, because the infection of HEV71 is directly related to its endocytosis. Therefore, the dynamic parameters (force and wrapping time etc.) of the HEV71 invagination process at its initial infection can provide the missing information to understand the viral infection mechanism, which will pave the way for the future diagnosis and treatment of HMFD.

As a result of the characteristics of molecular epidemiology and high rate of neurological complications of HEV71 virus, no effective vaccine is currently available, and treatments are only symptomatic. These conditions make it necessary to perform experimental operations related to live HEV71 virus in biosafety level 2 (BSL‐2) facilities,^9^ an inconvenience, considering the disposal of samples and use of instruments. As an alternative, virus‐like particles (VLPs) are empty particles consisting of viral structural proteins without viral genetic material. They are similar to the authentic virus structurally and can mimic authentic virus to elicit strong and broad immune responses; therefore, they are noninfectious and safe for experimentation in ordinary labs.^10^ Many types of VLPs have been expressed and applied for clinical research, such as Influenza,^11^ Severe Acute Respiratory Syndrome (SARS) coronavirus^12^ and Hepatitis B virus,^13^ and to investigate the interactions between viruses and cells by colocalization and single‐particle tracking.^14^ Here, we use force tracing technique to directly record the endocytic force and wrapping time of single VLPs via cell membranes. Combining with simulation, we reveal the dynamic process of single VLPs invagination, which provides critical hints for further understanding the mechanism of viral infection.

## 2 Results

### 2.1 The Examination of VLPS

**Figure 1:**
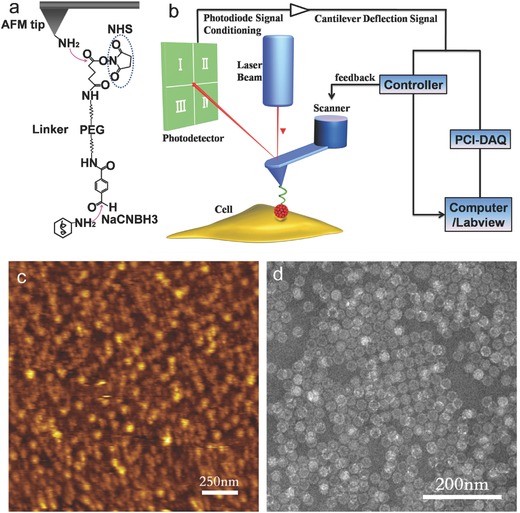
Schematic illustration of the principle and process of force tracing. a) The scheme of a functionalized AFM tip. The virus was covalently coupled to AFM tips via a heterobifunctional PEG linker. b) The schematic setup of force tracing based on AFM and PCI‐DAQ. c) Image of silicon with tethered viruses. d) The TEM image of the VLPs of HEV71 viruses shows that the VLPs are well‐dispersed and uniform.

In this study, African green monkey kidney cells (Vero) were used to study the mechanism of virus because of their relatively high sensitivity of EV71.^5^ The VLPs of HEV71 virus were expressed and purified through constructing a recombinant baculovirus that coexpresses HEV71 P1 and 3CD proteins by a Bac‐to‐Bac Baculovirus Expression System (Figure S1, Supporting Information). In order to examine whether the VLPs can be used to replace the authentic HEV71 virus for infection in Vero cell, we used the anti‐HEV71 monoclonal antibody for locating virus infection (Figure S2, Supporting Information). These results demonstrated that HEV71 VLPs do not alter the entry property and can be used for further research. Then, the purified VLPs were covalently conjugated onto an atomic force microscopy (AFM) tip via a heterobifunctional polyethylene glycol (PEG) linker and the VLP could not enter the cytoplasm, thus the cell viability remained constant after performing force tracing curves. The tip modification is shown in **Figure** **1**a. To verify the viral density on the AFM tip, the silicon surface was functionalized with virions under the same conditions as those on the AFM tip. The AFM image shows that the silicon surface is covered with a dense monolayer of about 120 virions per square micrometer (Figure 1c). The diameter of a single virion is 26 ± 2 nm which is measured by transmission electron microscopy (TEM) (400 particles were analyzed), as shown in Figure 1d. Taking into account a tip radius of about 20–30 nm, the density of virions on the silicon surface confirms that, in most cases, only a single virus particle was attached on the tip apex for force measurement, which is suitable for investigating the single viral entry.

### 2.2 Schematic Illustration of Force Tracing

We used the “force tracing” technique based on AFM to follow the invagination of HEV71^15^ (setup is shown in Figure 1b). The AFM tip was engaged to the contact point between the AFM tip and cell surface (details are shown in Figure S3, Supporting Information). A beam of laser is reflected by the AFM cantilever. The photodetector detects the laser position and records the change of cantilever location. Upon the external force, the cantilever would deflect and the vertical change of cantilever was acquired by PCI‐DAQ card that can easily monitor the fast process down to 1 µs and be suitable for the recording the process of viral entry into living cells.

### 2.3 Force Tracing Curves

**Figure 2:**
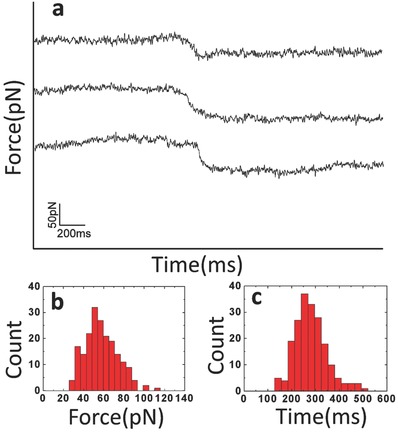
Force tracing curves based on AFM. a) Typical force tracing curves showing viral invagination via cell membranes. b) Distribution of force for cellular uptake of virus (*n* > 190 from about 2000 force tracing curves). c) Distribution of time for viral invagination via cell membranes.

**Figure** **2**a represents a typical force tracing curves. The tracing signal begins from the left. At the beginning, the AFM tip‐tethered virions would stay on the cell surface. While the membrane invaginates to form the vesicle packing virus, the AFM tip will bend downward and a force signal could be detected. After the endocytosis force and bending force of AFM cantilever reach equilibrium, the virus cannot move further into the cell. The abscissa (*X* axis) represents the time of force tracing, and the ordinate (*Y* axis) represents the deflection of the AFM tip corresponding to the force. The force signal is based on the detection of small shifts of the cantilever‐deflection signal that occurs when a tip‐tethered virus is undergoing cell endocytosis.

The core interest of this study focuses on measuring the force and time of viral invagination from the force tracing curves. The force of virus infection ranges from 40 to 80 pN with a maximum distribution of 58 ± 16 pN, as shown in Figure 2b. This result indicates that the invagination of virus is driven by a force of about 60 pN, a value that cannot be obtained from fluorescence‐based single particle tracking. The time distribution of viral invagination (duration) is 279 ± 68 ms (Figure 2c), which is much more fast than we expected.

### 2.4 Control Experiment

**Figure 3:**
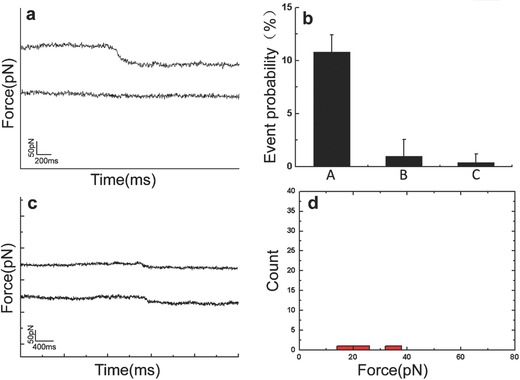
Control experiments. a) Typical force tracing curves (upper) of virus entry. No signals (lower) were observed when blocked with cytochalasin B. b) The probability of tracing curves with force signal under different conditions, including (A) (functionalized tip on cell surface without blocking), (B) (functionalized tip on cell surface after blocking with CB), (C) (clean tip without being modified with viruses on cell surface). Values are represented by mean ± standard deviation. c) Force tracing curves observed from 1000 curves with force signals was about 0.3% and the force value of about 20 pN. d) The force distribution of force tracing curves in the control experiment (*n* = 3 that were chosen from 1000 force tracing curves).

**Figure 4:**
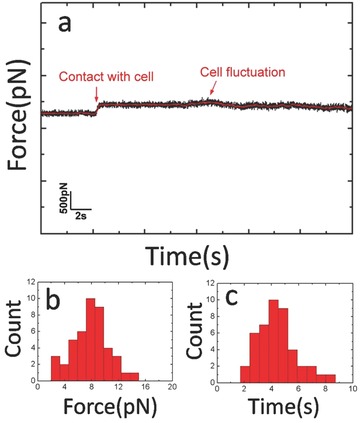
Fluctuation of living cells. a) Force tracing curves, while the clean AFM tip touched the cell membranes with a force of about 20 pN. b) Force distribution caused by cell membrane fluctuation. c) Time distribution of cell fluctuation.

To confirm the specificity of the force tracing events, a series of control experiments were performed. **Figure** **3**a shows the typical curves before (lower) and after (upper) blocking with reagents. After injecting cytochalasin B (CB), a cell‐permeable mycotoxin, into the AFM liquid chamber during force tracing measurement, most of the force signal disappeared, and the probability of tracing curves with force signal decreased from 10.8% (Figure 3b(A)) to 1% (Figure 3b(B)). We also engaged force tracing curves on the Vero cells by the clean AFM tip (without being modified with VLPs), and the probability of force tracing curves with force signals was about 0.3% (Figure 3b(C)). The force value of force tracing curves engaged by the clean tip was obviously decreased to about 20 pN (Figure 3c,d), which is much smaller than that from a virus‐tethered tip (58 ± 16 pN). At last, we measured the fluctuation of the living cells, and the force signal was easily distinguished from the fluctuation of living cells (**Figure** **4**). To measure the fluctuation of a living cell, the clean AFM tip was approached to the cell surface and gently touched the cell membrane with a force of about 20 pN. Then the feedback system was switched off and the cantilever moved free. The fluctuation of the cell could be detected (Figure 4a). The long‐lasting experiment for recording the cell fluctuation with clean AFM tip is shown in Figure S4 (Supporting Information). The force caused by cell membrane fluctuation is much smaller than the endocytosis force (Figure 4b). We also noticed that the time of cell membrane fluctuation was at the level of several seconds (Figure 4c), which is larger than that from the endocytosis force in Figure 2a. These control experiments confirm that the force tracing signals were caused by the viral invagination.

### 2.5 Virus Displacement

The VLPs were modified on the AFM tip via a PEG linker about 30 nm long. The viral invagination causes the downward bending of the cantilever as well as the extension of the PEG linker. Hence, the virus displacement *D* is equal to the bending distance *d* of the cantilever and the stretching length *x* of the PEG linker

(1) D=d+x

The force–extension curves of PEG can be most appropriately described by the extended worm‐like chain model that characterizes the force (*ƒ*)‐dependent stretching behavior by the equation

(2) $$\frac{FL_{p}}{k_{B}T}\;\;=\;\;\frac{1}{4}{\left(1-\frac{x}{L_{0}}+\frac{F}{K_{0}}\right)}^{-2}-\frac{1}{4}+\frac{x}{L_{0}}-\frac{F}{K_{0}}$$

where *k*_B_ is the Boltzmann constant, *T* is the absolute temperature, *L*_p_ is the persistence length, *K*_0_ is the enthalpic correction, *x* is the extension, and *L*_0_ is the contour length. The persistence length is 3.8 ± 0.02 Å, and the enthalpic correction is 1561 ± 33 pN, as reported.^16^ Given that the PEG unit length is 4.2 Å and the terminus is 5.25 Å, the total estimated contour length for PEG of 76–77 mers is about 326 Å. The bending distance of the cantilever can be calculated from Hooke's law by the equation

**Figure 5:**
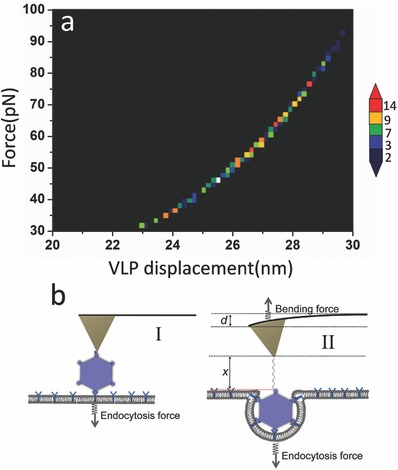
Detecting virus displacement via force tracing curve. a) The sum of linker extension and cantilever deflection according to the force caused by viral endocytosis. b) The scheme of viralinvaginationvia cell membranes.

(3) F=kd

where *F* is the force measured from the force tracing curve, and *k* is the spring constant of the cantilever. From the above equations, it is clear that the viral displacement is directly correlated to the force we measured. **Figure** **5**a shows force–displacement 2D histogram. The maximum distribution of displacement is around 27 nm, which is similar to the viral size (Figure 5b). Given the time duration is about 280 ms, the velocity of viral internalization is about 0.1 µm s^−1^, which is similar to the velocity of viral movement detected by the fluorescence microscopy.^17^ In our work, the method has advances in probing viral endocytosis with nanometer and microsecond resolution.

### 2.6 Simulations

**Figure 6:**
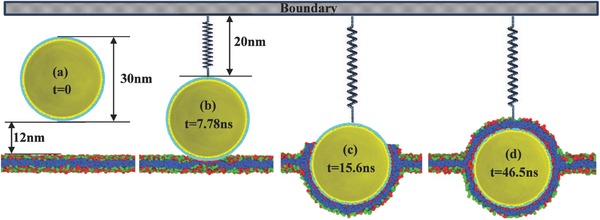
Dynamic trajectories of nanoparticle endocytosis by cell membranes.

We further investigate the atomic mechanisms of viral entry by using molecular dynamic simulation (details are shown in Figure S5, Supporting Information). Initially, a sphere rigid nanoparticle (represents the virus) with diameter of 30 nm is created and put 27 nm above the bilayer membrane. The system is relaxed for 10 ns, while the virus is fixed at its initial position. Then through steered molecular dynamics,^18^ the nanoparticle is pulled down toward the bilayer membrane with a constant pulling velocity 0.1 *r*_c_/τ until it reaches to the surface of the bilayer membrane. In order to measure the force required by the cell membrane to endocytose the nanoparticle, the nanoparticle is assumed to attach to a virtual spring on the boundary with its spring constant as 0.5 *k*_B_ *T*/σ^2^ and its equilibrium distance *R*_0_ = 20 nm as shown in **Figure** **6**. At the beginning of the simulation, the membrane is under zero tension with the lipid per area *A*_0_ = 1*r*_c_^2^. During the simulations, in order to keep the membrane surface under zero tension, the size of the box in the *X*–*Y* plane is tuned to maintain the zero‐tension condition. For example, if the new area per lipid *A*_b_ is greater than *A*_0_, the simulation box will be compressed in the *X*–*Y* plane until *A*_b_ = *A*_0_, while if *A*_b_ < *A*_0_ the box will be stretched in the *X*–*Y* plane until *A*_b_ = *A*_0_. Meanwhile, the box length in the normal direction of the membrane will change correspondingly to keep the box volume fixed. It has been widely revealed that the receptor on the cell membrane could facilitate the endocytosis process of exotic nanoparticles.^19^, ^20^ Before contact with the virus, the receptors in the cell membrane are assumed to be uniformly distributed. Thanks to the strong attraction between the virus and receptors on the bilayer membrane, once the contact between virus and cell membrane starts, the receptor density within the contact area increases and the receptors in the neighborhood of the contact region are driven to the contact zone by diffusion.^21^ The relatively large nanoparticles compared with the membrane thickness (5 nm) may easily increase the adhesion area and strength with the membrane which further increase the diffusive motion of receptors toward virus.^20^ Our simulation shows that the nanoparticle is quickly (7.82 ns) endocytosed by the cell membrane after attaching onto the membrane (Figure 6b,c). Previous studies have shown that there is an optimal nanoparticle radius for endocytosis by cells resulting from the competition between the thermodynamic driving force and receptor diffusion kinetics.^21^ In this study, the nanoparticle cannot be fully engulfed due to its large size. The upper surface of the virus is eventually covered by the receptors traveling over a longer distance; hence, a longer wrapping time (30.9 ns) is required for the process (Figure 6c,d).

## 3 Discussion

HEV71 is a major pathogen, which causes outbreaks of HFMD. The molecular epidemiology, evolution, and molecular genetics of HEV71 have been studied widely,^1^ however, the dynamic process of viral invagination is still unclear because of the limitation of current approaches. HEV71 is a small and round virus with the shape of regular icosahedron and the size of ≈30 nm, which is suitable for tip functionalization and particle tracking analysis. By force tracing with constant piezoceramics position, the process of viral invagination via cell membranes was recorded until it reaches the balance between endocytosis force and pulling force of AFM cantilever. The force tracing technique occupies several advantages: (i) It allows the single virion investigation, which provides a potent approach to study viral behaviors at the single particle level; (ii) The force of viral invagination is obtained, which is unavailable by real‐time fluorescence microscopy,^17^, ^22^ (iii) It enables to extremely fast record the viral invagination at the microsecond level and provide detailed dynamic information, which is unachievable by other current techniques.

In this particular study, we measured the mechanical pulling force of HEV71's invagination at high sensitivity (≈60 pN). The endocytosis of virus involves a large number of proteins with various functions that are cooperated to generate the mechanical pulling force; therefore, knowing how much force is needed to wrap the virus is essential to understand how the cooperated proteins work. After injecting cytochalasin B, almost no force tracing signal was detected, which confirms that the mechanical pulling force is generated by actin filaments.^6^

Figure 5a shows that the viral displacement is consistent with the viral size, which implies that the virus was partially wrapped (Figure 5b). The duration for viral invagination is ≈280 ms, which is shorter than the theoretical predicted wrapping time of nanoparticle with a diameter of about 30 nm.^21^ Our simulation shows that the virus can reach a partially wrapped state very fast (Figure 6b,c), then it takes almost four times as long (30.9/7.82) for the membrane traveling over a longer distance to completely wrap the virus (Figure 6c,d). Although the time scale between experiments and simulations is always disparate,^23^ the simulations in this work provides a hint for understanding why the viral internalization time we detected is shorter than theoretical prediction. In this work, the time we detected is that it takes for the HEV71 to be partially wrapped; hence, it is much shorter than the theoretical predication (2 s).^21^ According to the process provided by simulation, it may take a much longer period time to completely wrap the virus. Combining the experiment with the simulation, it is speculated that the wrapping of HEV71 occurs in two phases. First, the wrapping of virus is dynamically trapped at a partially wrapped state rapidly, and the main mechanical pulling force is generated in this step. Then, the upper surface of the virus is covered by the receptors traveling over a long period of time.

## 4 Conclusions

In summary, we have successfully recorded the process of single‐virus particle invagination via cell membranes by force tracing technique. The values of force, duration, and velocity for membrane wrapping of single HEV71 were measured in this study. A series of control experiments demonstrated that the force signal was resulted from endocytosis. Combining the experiment with the simulation, the mechanism of membrane wrapping of virus is clearly revealed. This work provides the dynamic parameters (force and wrapping time etc.) of the HEV71 invagination process at its initial step, which is helpful for understanding the infection of HEV71.

## 5 Experimental Section

*Immunofluorescence Assays and Pharmacological Inhibition*: Vero cells (1 × 10^5^ per well) were seeded and cultured in glass‐bottomed petri dishes (NEST, China) 1 d prior to exposure to the monoclonal antibody and/or inhibitor chlorpromazine (CPZ) (Sigma‐Aldrich). Vero cells were infected with HEV71 viruses (multiplicity of infection, MOI = 2) and VLPs (10 µg mL^−1^) for 3 h at 37 °C. After a wash with phosphate‐buffered saline (PBS), the cells were fixed with methanal and then incubated with the primary antibody MAb8430 (1:100, Millipore), a mouse monoclonal antibody that specifically recognized HEV71 and EV70. Fluorescein isothiocyanate (FITC) labeled goat antimouse IgG antibody (1:5000, Bioon) was as second antibody for examining the fluorescence signals, and the nuclei were stained using Hoechst 33 258 (Beyotime). Pharmacological inhibition experiments were performed during HEV71 infection: cells were incubated with CPZ (30 × 10^−6^ m) for 2 h at 37 °C, then were infected with HEV71 viruses or VLPs. The unbound virus and drugs were washed with PBS. Signals were detected with using the Ultra View VOX (PerkinElmer) confocal system with an inverted microscope (Nikon).

*Modifying Glass Slide with (3‐Aminopropyl)triethoxysilane (APTES)*: APTES‐glass slide substrate was prepared as described.^24^ Briefly, a desiccator was purged with argon for 2 min, and 30 µL APTES (99%) and 10 µL *N*,*N*‐diisopropylethylamine (DIPEA) (99%) were respectively placed into one small container at the bottom of the desiccator. Subsequently, the desiccator was purged with argon for another 2 min. Glass slides were placed into the prepared desiccator, and then the desiccator was sealed off after purging for another 3 min, leaving the glass slides exposed to APTES vapor for 1 h. After this exposure, the containers with APTES and DIPEA were removed, the desiccator was purged again, and the treated glass slides were stored in the sealed desiccator prior to use.

*Cell Culture*: Vero cells were obtained from the Shanghai Institutes of Biological Sciences. The cells were cultured on APTES‐glass slides in Dulbecco's modified Eagle's medium (DMEM) containing 10% fetal bovine serum, 100 U mL^−1^ penicillin, and 100 µg mL^−1^ streptomycin at 37 °C with 5% CO_2_. Usually, the cells need to be cultured for 2 or 3 d to achieve 75% confluence on the glass slide.

*Virus Infection and Purification of VLPs*: Hi5 cells were infected with the recombinant baculovirus Ac‐P1‐3CD at a MOI of 5. The infected cells were collected at 72 h postinfection, and cell supernatant was removed by centrifugation at 4000 g for 20 min. Cells were resuspended in lysis buffer (0.01 m Tris‐HCl, 0.001 m EDTA, 0.01 m 2‐mercaptoethanol, 100 g L^−1^ NP‐40, pH 7.4) at 4 °C for 1 h, then lysed by sonication for 3–5 min and centrifuged at 10 000 *g* (12150‐H rotor, Sigma) for 30 min. The supernatants were filtered through 0.22 µm filter (Millipore) and subsequently centrifuged at 35 000 rpm at 4 °C for 3 h (Ti70 rotor, Beckman). The pellets were resuspended in TE buffer (0.01 m Tris‐HCl, 0.001 m EDTA, pH 7.4), filtered through 0.22 µm filter and loaded onto the sucrose gradient (10%, 20%, 30%, 40%, 50%, 60%, w/v). After ultracentrifugation at 28 000 rpm (SW40 rotor, Beckman) for 4 h, the white band between the interfaces of 40%–50% sucrose was collected and diluted in TE buffer. Sucrose was removed by centrifugation at 35 000 rpm at 4 °C for 3 h (Ti70 rotor, Beckman). Purified viruses were resuspended in 0.01 m PBS and stored at 4 °C for transmission electron microscopy analyses.

*Tip Modification*: AFM tips (MSCT, D‐tip, Veeco, Santa Barbara, CA) were functionalized with APTES in a manner similar to the preparation of APTES‐glass slides, as described above. The cantilevers were cleaned in a UV cleaner and vapor‐treated with APTES. Subsequently, PEG crosslinker (benzaldehyde‐PEG76‐NHS, FW∼3962, SensoPath Technologies, Bozeman, MT) was conjugated in triethylamine and trichloromethane as described.^25^ After drying with argon, the tips were then immersed in a mixture of 100 µL HEV71 in PBS and 4 µL 1 m NaCNBH_3_. After functionalization for 60 min, 10 µL 1 m ethanolamine was added to the solution in order to passivate the unreacted aldehyde groups. Then the AFM tips were washed with PBS three times and stored at 4 °C.

*Force Tracing Measurements*: Force tracing curve was acquired using the AFM 5500 (Agilent Technologies, Chandler, AZ) in DMEM at 37 °C without controlling CO_2_ concentration. Before performing the experiment, the AFM tip was usually stabled in DMEM for about 1 h. The small shifts of the cantilever‐deflection signal were collected by a 16‐bit DA/AD card (PCI‐6361e, National Instruments). We performed about 2000 force tracing curves on about 500 cells. The time for performing a typical force tracing curve is 10 min. The viral invagination probability is about 10.8%. The blocking experiments were performed by the addition cytochalasin B (final concentration of 1 µg mL^−1^) into the culture medium, respectively. Thousand tracing curves were recorded at different positions on about 200 cells. The deflection sensitivity of the photodetector and the spring constant of the AFM tip were determined according to a previous report.^26^ The average spring constant was about 0.03 N m^−1^. The sampling rate is 20 kHz and the data were collected with the low‐pass filter of 100 Hz to eliminate high frequency noise from the electronics and environment.

## Conflict of Interest

The authors declare no conflict of interest.

## Supporting information

Supplementary
