# Supplementary material for: The Process of Wrapping Virus Revealed by a Force Tracing Technique and Simulations
Source: Adv Sci (Weinh). 2017 Apr 26;4(9):1600489. doi: 10.1002/advs.201600489 (PMC5604396; doi:10.1002/advs.201600489)
Supplement: Supplementary file 1 — Supplementary [file ADVS-4-na-s001.pdf]

Copyright WILEY-VCH Verlag GmbH & Co. KGaA, 69469 Weinheim, Germany, 2017.

ADVANCED SCIENCE Open Access

Supporting Information

for *Adv. Sci.,* DOI: 10.1002/advs.201600489

The Process of Wrapping Virus Revealed by a Force Tracing Technique and Simulations

Yangang Pan, Fuxian Zhang, Liuyang Zhang, Shuheng Liu, Mingjun Cai, Yuping Shan, Xianqiao Wang,* Hanzhong Wang,* and Hongda Wang**

Title: The Process of Wrapping Virus Revealed by a Force Tracing Technique and Simulations

Authors: Yangang Pan[1#], Fuxian Zhang[2#], Liuyang Zhang[3#], Shuheng Liu[1], Mingjun Cai[1], Yuping Shan[1,4]*, Xianqiao Wang[3,]*, Hanzhong Wang[2,]* and Hongda Wang[1,5,]*

Keyword: Force Tracing, HEV71, Invagination, Single particle simulation, Wrapped

Supporting Information

The Process of Wrapping Virus Revealed by a Force Tracing Technique and Simulations

Yangang Pan[1#], Fuxian Zhang[2#], Liuyang Zhang[3#], Shuheng Liu[1], Mingjun Cai[1], Yuping Shan[1,4], Xianqiao Wang[3,]*, Hanzhong Wang[2,]* and Hongda Wang[1,5,]**

[#]These authors contributed equally

Dr. Y. Pan, Dr. Y. Shan, MS. S. Liu, MS. M. Cai, Prof. H. Wang State Key Laboratory of Electroanalytical Chemistry, Changchun Institute of Applied Chemistry, Chinese Academy of Sciences, Changchun, Jilin 130022, P.R. China.
E-mail: hdwang@ciac.ac.cn

Prof. H. Wang, University of Chinese Academy of Sciences, Beijing 100049, P.R. China.

Dr. F. Zhang, Prof. H. Wang, Key Laboratory of Special Pathogens and Biosafety, Center for Emerging Infectious Diseases, Wuhan Institute of Virology, Chinese Academy of Sciences, Wuhan, 430071, China.
Email: wanghz@wh.iov.cn

Dr. L. Zhang, Prof. X, Wang College of Engineering, University of Georgia, Athens, GA 30602, USA
Email: xqwang@uga.edu

Dr. Y. Shan, School of Chemistry and Life Science, Advanced Institute of Materials Science, Changchun 130012, China
E-mail: shanyp@ciac.ac.cn

Keywords: force tracing, HEV71, internalization, single particle simulation, wrapped

1. Construction of the recombinant bacmid (BAC-P1-3CD).

Based on previous studies[1], we construct the recombinant baculovirus (Ac-P1-3CD) carrying P1 protein and 3CD protein by Bac-to-Bac baculovirus expression system. The Polymerase Chain Reaction (PCR) product of P1 was inserted into downstream of the pH promoter of the transfer vector pFastBacDual (Invitrogen), then 3CD was inserted to the downstream of the p10 promoter to construct the donor plasmid pFastBac-P1-3CD. The recombinant bacmid (Bac-P1-3CD) (Figure S1) was generated through translocating the donor plasmid to the AcMNPV bacmid (Ac-BAC) in the presence of transposases. The verified bacmid DNA was transfected into Sf9 cells to obtain the recombinant virus (Ac-P1-3CD).

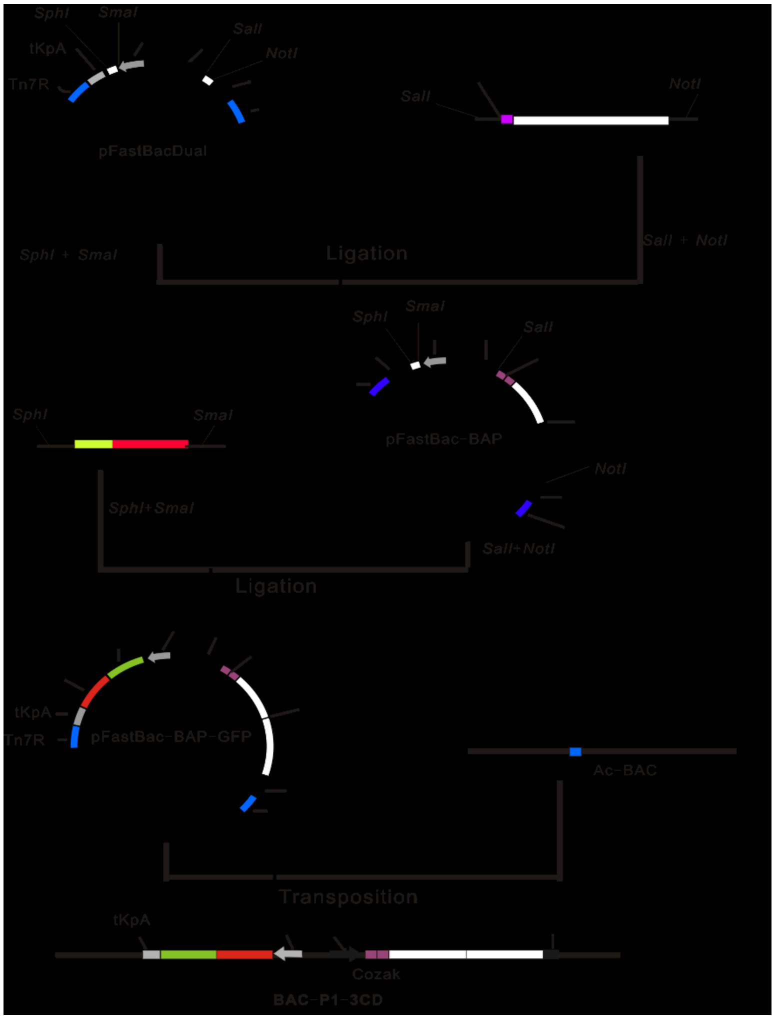

Figure S1. Construction of the recombinant bacmid (BAC-P1-3CD).

2. The examination of VLPS.

HEV71 VLPs were produced by infecting Hi5 cells with the recombinant baculovirus Ac-P1-3CD. The infected cells were harvested at 72h post-infection, and the cell lysate was collected to purify the VLPs by sucrose density gradient centrifugation. The VLPs were examined by immunofluorescence assay (IFA) transmission and electron microscope (TEM). The green fluorescence particles were distributed in the cytosol and on the membrane surface of VLP-incubated Vero cells (Figure S2c), which was consistent with the result of the immunofluorescence assay of HEV71 virus (Figure S2a). To further confirm the HEV71 VLPs infection in Vero cells, we used chlorpromazine (CPZ) as an inhibitor to inhibit the internalization process of the HEV71 viruses and VLPs. Figure S2b and S2d show that VLPs

endocytosis was inhibited largely. In the control experiment, no green fluorescence particle was observed in the cytosol of cells (Figure S2e).

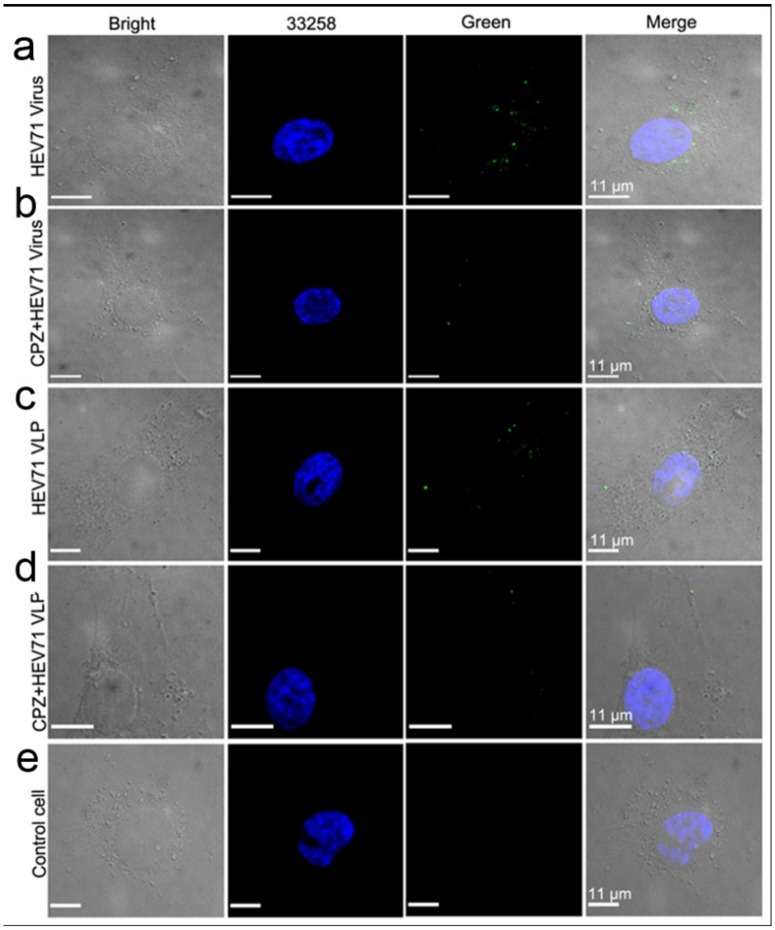

Figure S2. The immunofluorescence assay (IFA) and control experiments confirm the VLP infection in Vero cells. (a, c and e) IFA of HEV71 viruses and VLPs in Vero cells. Vero cells were infected with the indicated viruses, and the fluorescence intensity was examined under the confocal microscope. Uninfected Vero cells served as negative control. Green fluorescence indicates HEV71 viruses, blue signal shows the nuclei stained with Hoechst. a: HEV71 virus; c: VLPs; e: Control cell. (b, d) Pharmacological inhibition during virus infection. Vero cells were treated with CPZ prior to infect with the indicated viruses. After incubation with the monoclonal antibody MAb8430 and FITC-labeled second antibody, the fluorescence signals were detected by fluorescence microscope. b: HEV71 virus; d: VLPs.

3. Engaging the tip

African green monkey kidney (Vero) cells, which can be infected by HEV71, were used to study the mechanism of virus entry. In this particular study, we prepared a dense monolayer of Vero cells on the glass surface functionalized with (3-Aminopropyl) triethoxysilane (APTES). Usually, the cells were cultured for 2 or 3 days on the glass slide. Before performing Force Tracing, a CCD camera was used to locate the AFM tip on the top of the Vero cell monolayer (Figure S3a). To determine whether the tip can be approached to the cell surface with high precision, we approached the AFM tip to contact point at a rate of about 50 nm/s repeatedly (Figure S3d). The results show that relative position between the cell and the tip has remained within ± 7nm range (Figure S3e), which illustrates that this method engage the AFM tip to the living cell surface with high precision. Then, we engaged the functionalized AFM tip, and we did force-distance curves on the living cell so that we can locate the contact point between the AFM tip and cell surface, as illustrated in Figure S3f. We took advantage of the feedback system to approach the AFM tip to the contact point between the AFM tip and cell surface at a rate of about 50 nm/s and then turned off the feedback system (Figure S3g I). As a result, the AFM tip suspended on the cell surface, as shown in Figure S3c and S3g II. Upon viral internalization, the cantilever of the AFM tip bent downward, and the force signal was recorded *via* a Force Tracing curve, as shown in Figure S3d and S3g III. The contact point can be accurately reached repeatedly.

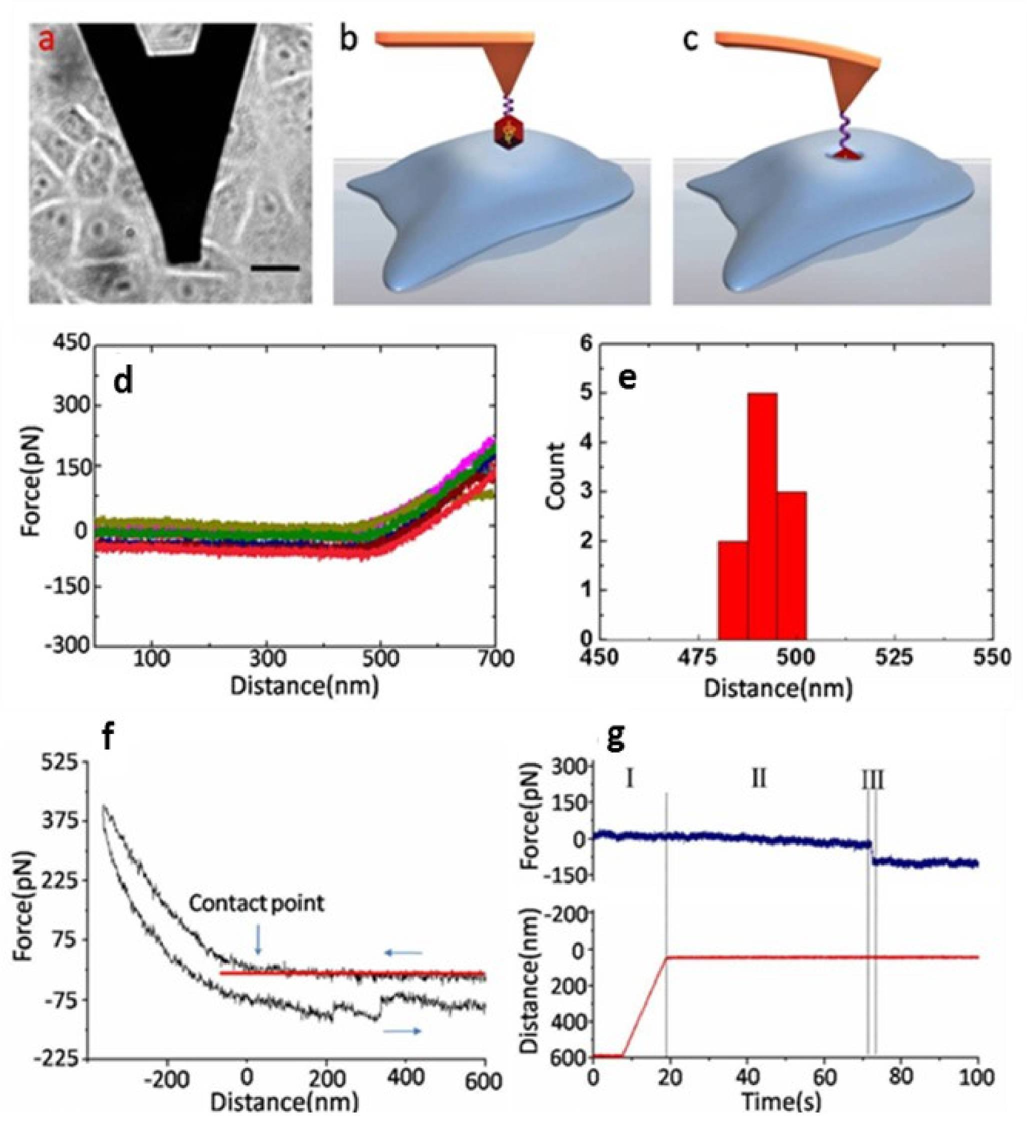

Figure S3. Force Tracing engaging and the contact point. (a) The optical image of AFM cantilever located above the dense monolayer of living Vero cells. The scale bar is 20 µm. (b and c) Diagram of Force Tracing process. (b) The AFM tip was tethered with virus suspended on the living cell surface. (c) The lower solid AFM tip represents tethered-virus entry into living Vero cell through endocytosis, which induces the signal of Force Tracing. (d) The AFM tip was approached to contact point at a rate of about 50 nm/s repeatedly for 10 times. (e) Distribution of distance for approaching the AFM tip to the contact point (n=10), which shows that the variation of contact points is within ± 7nm. (f) Force curves on the cell surface to find the contact point. (g) Full Force tracing curve based on AFM to show how we get the data. The upper trace

represents the deflection of the AFM tip over time, and the lower trace shows the length change of piezoelectric ceramic over time.

4. The long lasting AFM experiment.

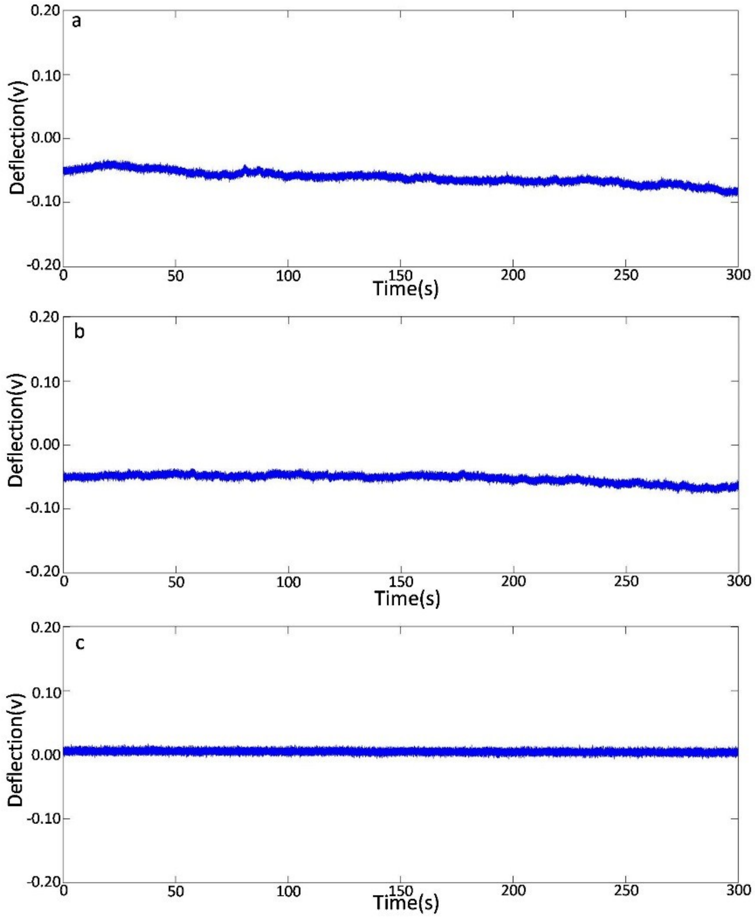

Figure S4. Fluctuation of living cells (a) Long lasting Force Tracing curves while performing Force Tracing curves with clean AFM tip. (b) Long lasting Force Tracing curvesafter adding cytochalasin B. (c) Force Tracing curves with the AFM tip away from the cell membrane.

5. Computational Model and Methodology

Simulations were based on dissipative particle dynamics (DPD), a mesoscopic coarse-grained simulation method for soft matter and biomembrane systems[2-6]. In DPD simulation, a small group of atoms is treated as a single bead located at the center of mass of the group. Beads *i* and *j* interact with each other via a pairwise force consisting of a conservative force F_{ij}^{C} representing excluded volume effect, a dissipative force F_{ij}^{D} representing viscous drag between moving beads, and a random force F_{ij}^{R} representing stochastic impulse. Both F_{ij}^{D} and F_{ij}^{R} act together as a

thermostat for beads. Similar to molecular dynamics simulation, time evolution is also governed by the Newton's equation of motion. The total force on bead i can be expressed as

$$F_i = \sum_{i \neq j} \left(F_{ij}^C + F_{ij}^D + F_{ij}^R\right) = \sum_{i \neq j} \left(a_{ij}\omega(r_{ij})\hat{r}_{ij} - \gamma\omega^2(r_{ij})(\hat{r}_{ij} \cdot v_{ij})\hat{r}_{ij} + \sigma\omega(r_{ij})\zeta_{ij}\Delta t^{-1/2}\hat{r}_{ij}\right)$$

where a_{ij} is the maximum repulsive force, r_{ij} the distance, $\hat{r}_{ij}$ the unit vector and v_{ij} the relative velocity between beads i and j; ζ_{ij} denotes a random number with zero mean and unit variance, and

$$\omega(r_{ij}) = \begin{cases} 1 - \frac{r_{ij}}{r_c}, & r_{ij} < r_c \\ 0, & r_{ij} > r_c \end{cases}$$

is a normalized distribution function, r_c being the cutoff radius; γ and σ are parameters related to each other as $\sigma^2 = 2\gamma k_B T$, $k_B T$ being the unit of energy. The standard values $\sigma = 3.0$ and $\gamma = 4.5$ are used in our study[3-5]. The mass, length, and time scales are all normalized in the DPD simulations, with the unit of length taken to be the cutoff radiusr_c, the unit of mass to be that of the solvent bead, and the unit of energy to be $k_B T$. All other quantities are expressed in terms of these basic units. The reduced DPD unit can be converted to SI unit by examining the membrane thickness and the lipid diffusion coefficient. The simulated value of the bilayer thickness is $5r_c$ and the effective time scale of simulation can be determined from the simulated lateral diffusion constants of the lipid bilayer[4]. The DPC bilayer thickness is about 4 nm with a diffusion coefficient around 5 $\mu m^2 s^{-1}$,[7] by comparison with typical experimental values, it can be shown that one DPD length unit corresponds to approximately 0.8 nm in physical unit and the time unit to $\tau = 24.32$ ps. The time step is taken as $\Delta t = 0.01\tau$. All simulations are based on LAMMPS package [8].

The dimension of the simulation box is $120r_c \times 120r_c \times 130r_c$. Periodic boundary conditions are adopted in three directions.The system contains a total of 5,329,858 particle, 5,113,858 water beads, 187,200 lipid beads and 28,800 nanoparticle beads, with a particle density of approximately 3[4]. The solvent beads are not shown for clarity. The simulation system consists of the bilayer membrane with 14,400 lipid molecules (50% of them are assumed as receptors), a sphere viruswith stiff hydrophobic core coated by a single layer of hydrophilic ligands. The lipid molecule is represented by a coarse-grained model proposed by Groot and Rabone [2], as shown in S5, with 3 lipid hydrophilic head beads and 10 lipid hydrophobic tail beads. The parameters

for repulsive interactions between different types of beads are detailed in Table 1. Within a lipid molecule, an elastic harmonic force,

$$F_{ij}^{S} = k_s(1 - \frac{r_{ij}}{r_s})\,\hat{r}_{ij}$$

is used to connect two consecutive beads, where k_s and r_s are the spring constant and equilibrium bond length respectively. Here we use $k_s = 100.0$, $r_s = 0.7r_c$ for lipid molecule[9]. Hydrophilic ligands are linked to each nanoparticle core bead with $k_s = 200.0$, $r_s = 0.7r_c$. The bending resistance of the lipid chain is represented as an additional force due to a harmonic constraint on two consecutive bonds,

$$F^{\theta} = -\nabla V_{bend} = -\nabla[k_{\theta}(\theta - \theta_0)^2]$$

where k_{θ}, θ and θ_0 are the bending constant, inclination angle and equilibrium angle respectively. For three consecutive lipid tail beads or three consecutive lipid head beads in a lipid molecule, we take $k_1 = 6$ and $\theta = 180^{\circ}$; for the last head-bead and the top tail-beads $k_2 = 3$ and $\theta = 120^{\circ}$; for the bottom two consecutive head beads and the first bead in each tail $k_3 = 4.5$ and $\theta = 120^{\circ}$[3, 4].

Table 1. Soft-repulsive interaction parameters a_{ij} between lipid molecules (**H**ead and **T**ail), receptor molecules (**H**ead and **T**ail), ligands (**L**), nanoparticle (**N**), and water (**W**).

	L_H	L_T	R_H	R_T	**L**	**N**	**W**
L_H	25	100	25	100	80	100	25
L_T	100	25	100	25	80	25	100
R_H	25	100	25	100	15	100	25
R_T	100	25	100	25	80	25	100
**L**	80	80	15	80	25	100	80
**N**	100	25	100	25	100	25	100
**W**	25	100	25	100	80	100	25

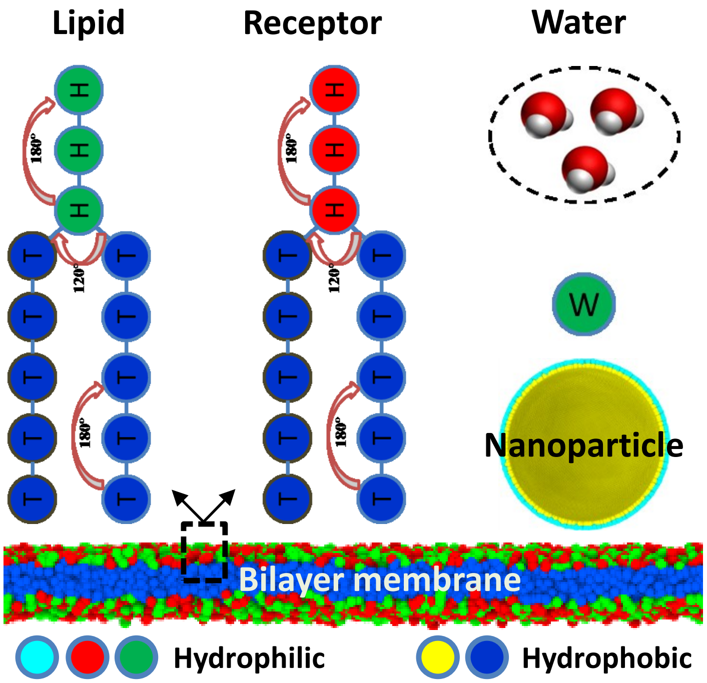

Figure S5. Schematic representation of the dissipative particle dynamics model. It includes the Coarse-grained model of lipid molecule (**L**), Coarse-grained model of receptor molecule (**R**), Coarse-grained model of water molecule (**W**), Bilayer membrane and nanoparticle (**N**). In the following figures, unless otherwise stated, the lipid tails are shown in blue while the lipid heads are shown in green. The receptor tails are shown in blue while the receptor heads are shown in red. The nanoparticle is shown in yellow. For clarity, the water is not shown in all figures.

References:

[1] a) Y. C. Hu, J. T. A. Hsu, J. H. Huang, M. S. Ho, Y. C. Ho, *Biotechnol. Lett.***2003**, *25*, 919; b) Y.-C. Chung, J.-H. Huang, C.-W. Lai, H.-C. Sheng, S.-R. Shih, M.-S. Ho, Y.-C. Hu, *World J. Gastroenterol.***2006**, *12*, 921.

[2] R. D. Groot, K. L. Rabone, *Biophys. J.***2001**, *81*, 725.

[3] L. Zhang, X. Wang, *Nanoscale***2015**, *7*, 13458.

[4] L. Zhang, M. Becton, X. Wang, *J. Phys. Chem. B***2015**, *119*, 3786.

[5] L. Zhang, X. Wang, *J. Phys. Chem. Lett.***2015**, *6*, 2530.

[6] L. Zhang, X. Wang, *J. Phys. Chem.* B 2016.

[7] J. C. Shillcock, R. Lipowsky, *Nat. Mater.* **2005**, *4*, 225.

[8] S. Plimpton, *J. Comput. Phys.***1995**, *117*, 1.

[9] M. Venturoli, B. Smit, M. M. Sperotto, *Biophys. J.* **2005**, *88*, 1778.
